# Discrepancy between surgeon and radiological assessment of ligation level of the inferior mesenteric artery in patients operated for rectal cancer—impacting registry-based research and surgical practice

**DOI:** 10.1186/s12957-021-02222-5

**Published:** 2021-04-13

**Authors:** Franciska Wikner, Peter Matthiessen, Karl Sörelius, Petter Legrell, Martin Rutegård

**Affiliations:** 1 Department of Radiation Sciences, Umeå University, Umeå, Sweden; 2 Department of Surgery, Faculty of Medicine and Health, Örebro University, Örebro, Sweden; 3 Department of Vascular Surgery, Rigshospitalet, Copenhagen, and Faculty of Health and Medical Sciences, University of Copenhagen, Copenhagen, Denmark; 4 Department of Surgical and Perioperative Sciences, Surgery, Umeå University, Umeå, Sweden; 5 Wallenberg Centre for Molecular Medicine, Umeå University, Umeå, Sweden

**Keywords:** Ligation level, High tie, Low tie, Anatomy, Computed tomography angiography, CT, Validation

## Abstract

**Background:**

The reliability of the registered ligation level of the inferior mesenteric artery (IMA) in the Swedish Colorectal Cancer Registry has been questioned. The primary aim of this study was to evaluate this parameter in the registry by comparing the registered ligation levels with a postoperative computed tomography angiography (CT-angiography) in patients operated for rectal cancer.

**Methods:**

Patients operated for rectal cancer at two Swedish university hospitals were prospectively included between December 2016 and December 2019. At the 1-year postoperative follow-up, an additional CT-angiography was performed and independently examined by two radiologists. The radiological assessment of the ligation level was compared to registry data, using different measures of agreement.

**Results:**

A total of 94 patients were included, 55 (59%) were men and 39 (41%) women. All patients underwent abdominal resection: conventional or robot-assisted laparoscopic surgery, *n*=56 (60%), or open resection, *n*=38 (40%). The ligation level as assessed on CT-angiography was high in 29 (31%) patients and low in 65 (69%). The registered level of ligation of the IMA and the radiological assessment of the CT-angiographies were consistent in 77/94 cases, demonstrating an 82% agreement and a sensitivity and specificity of 86% and 72%, respectively. The estimated Kappa value was 0.58, reaching 0.64 after prevalence bias adjustment.

**Conclusion:**

This study showed that CT-angiography can be used to evaluate the reliability of the registered ligation level in the Swedish Colorectal Cancer Registry. The demonstrated agreement between the registry and postoperative CT-angiography was moderate to good. This discrepancy impacts registry-based research using IMA ligation data and may ultimately influence surgical practice.

**Trial registration:**

Clinical Trials identifier NCT03875612

**Supplementary Information:**

The online version contains supplementary material available at 10.1186/s12957-021-02222-5.

## Introduction

Abdominal resection for rectal cancer includes ligation of the inferior mesenteric artery (IMA), either proximal (high ligation) or distal to the branching of the left colic artery (low ligation) [1, 2]. There are plenty of anatomic variations of the IMA and its branches [3]. Most commonly, the IMA courses downwards to the left of the aorta with the following branches and collaterals: (I) the left colic artery, supplying the descending colon, anastomosing with the middle colic artery of the superior mesenteric artery (via the peripheral mesenteric marginal artery of Drummond, the central mesenteric arc of Riolan, and the Villemin arcades); (II) the sigmoidal arteries supplying the sigmoid; and (III) the last and terminal branch is the superior rectal artery supplying the rectum, anastomosing with the middle and inferior rectal arteries (from the internal iliac artery) [3–6]. There is no consensus on the IMA ligation level, and the literature is conflicting. The potential benefits of high ligation are reduced risk of leaving metastatic apical lymph nodes and, in combination with ligation of the inferior mesenteric vein at the inferior border of the pancreas, obtaining a more mobile left colon for the colorectal or coloanal anastomosis [2, 7–10]. On the other hand, a low ligation is less invasive, might improve the collateral blood supply to the residual colon with a possibly lower risk of anastomotic ischemia and subsequent leakage, and carries lower risk of damage to the sympathetic autonomic nerve plexus adjacent to the proximal part of the IMA [11–13].

The Swedish Colorectal Cancer Registry (SCRCR) has nationwide coverage, and data are registered prospectively, including the ligation level of the IMA, as assessed by the operating surgeon [14]. However, the reliability of the registered ligation level has been questioned [11, 15]. In order to judiciously use the registry data with respect to the level of ligation of the IMA, further validation is warranted. Computed tomography (CT) angiography is a well-established diagnostic method to visualize the mesenteric arteries and might thus provide a reference against which the registry variable could be validated [16, 17].

The primary aim of this study was to evaluate the validity of the registered ligation level of the IMA in abdominal rectal cancer surgery in the SCRCR, as compared with the reference provided by radiological assessment from postoperative CT-angiography. Secondary aims were to describe the length of the remaining IMA stump after surgery, as well as to characterize the preoperative anatomy of the IMA, including the distance between the IMA and the aortic bifurcation.

## Methods

### Study design

This is a prospective validation study of the registered ligation level of the IMA in the SCRCR of rectal cancer patients who underwent abdominal resection with anterior resection, abdominoperineal excision, or Hartmann’s procedure. Two Swedish referral centers participated in the study: Umeå University Hospital and Örebro University Hospital. Patients were included during the period 7 December 2016 to 9 December 2019.

Patients were identified in the SCRCR, which has a 99% coverage of patients with rectal cancer in Sweden [14]. It is continuously validated against the National Cancer Registry for completion [1, 14, 15, 18]. The level of arterial ligation of the IMA as assessed by the operating surgeon was extracted for each patient from the SCRCR (Supplementary Figure 1), as well as clinical and demographic variables.

Rectal cancer was defined as an adenocarcinoma of the large bowel, where the aboral border of the tumor was at 15 cm or less from the anal verge, measured using rigid sigmoidoscopy. Minimally invasive surgery was defined as conventional laparoscopic or robot-assisted laparoscopic surgery.

All included patients were examined with a standard CT of the abdomen in venous phase, with an additional arterial phase of the mesenteric vessels (CT-angiography), at the standard postoperative 1-year follow-up; details are provided below. Inclusion criteria for the study were 1) operated rectal cancer, and 2) signed informed consent. Exclusion criteria were 1) no radiological follow-up, 2) CT examination without intravenous iodine contrast (due to, e.g., renal impairment, iodine contrast hypersensitivity, or known thyroid disease where radioiodine treatment could become relevant), 3) age <40 years, 4) pregnancy, 5) inability to understand patient information and give informed consent, 6) advanced disease where the patient is regularly undergoing radiological examinations to monitor progress, and 7) death before consent was requested.

### Implementation of CT-angiography

The CT-angiography of the mesenteric vessels was performed at the same time as the standard postoperative 1-year venous phase CT examination. CT systems used were Siemens Definition Flash (Siemens Healthineers, Erlangen, Germany), GE LightSpeed VCT (GE Healthcare, Milwaukee, WI, USA), GE Revolution CT (GE Healthcare, Milwaukee, WI, USA), and Siemens Definition AS (Siemens Healthineers, Erlangen, Germany). An angiography examination of the abdominal vessels was performed at each clinic and CT system, respectively; examination parameters were similar, providing a comparable radiation dose and image quality. Intravenous contrast enhancement with iodine contrast was administered using an individual dosage, by kilogram of body weight. Contrast was given via a high-pressure injector, where the injection rate was calculated using a dedicated computer program (OmniJect, GE). Details on CT manufacturer and respective survey parameters are summarized in Supplementary Table 1. Immediately after the arterial phase, the usual venous follow-up examination was performed.

### Evaluation of the arterial anatomy

**Fig. 1:**
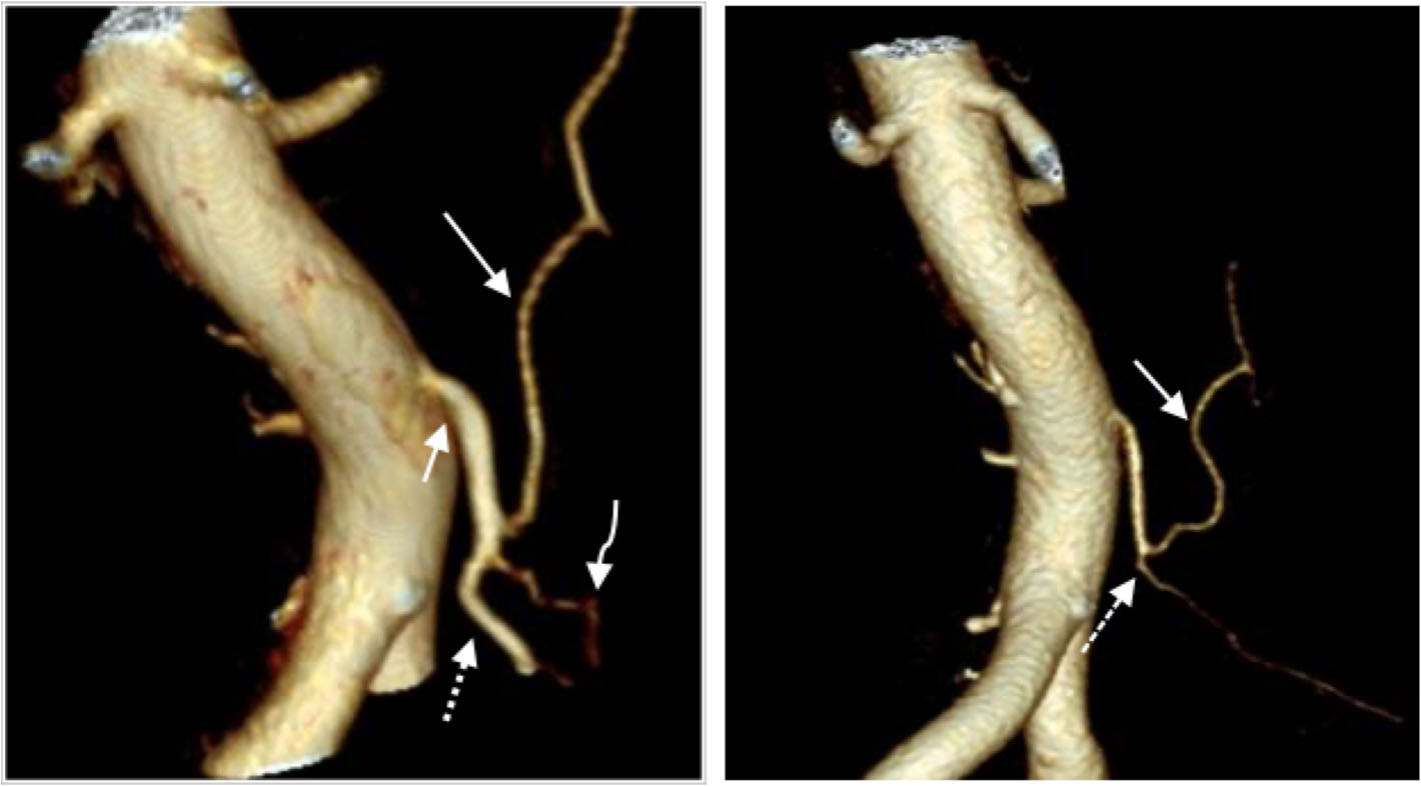
The left image shows the preoperative vascular anatomy with the inferior mesenteric artery (short arrow), the left colic artery (long arrow), the sigmoidal arteries (curved arrow), and the superior rectal artery (dashed arrow). The right image shows a postoperative image where the superior rectal artery cannot be found. The dashed arrow marks the ligation level caudally to the remaining left colic artery (solid arrow) and the sigmoidal arteries

**Fig. 2:**
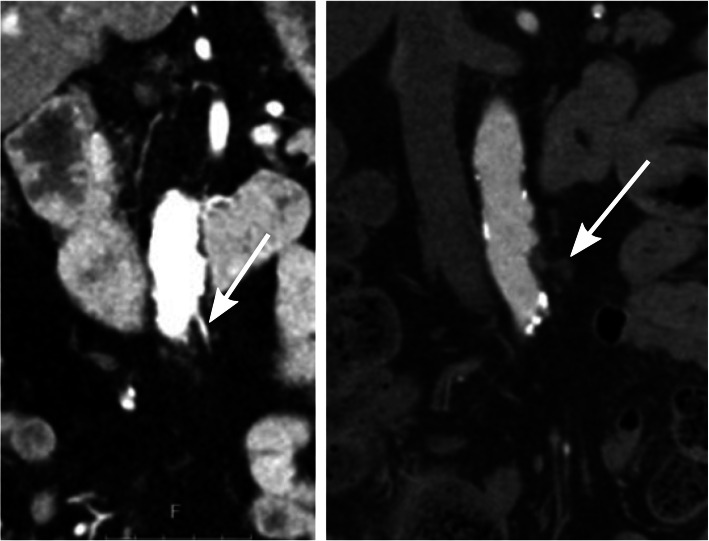
High ligation level where the inferior mesenteric artery preoperatively can be seen (the image to the left) and postoperatively where it cannot be found (the image to the right). The arrow in the postoperative image to the right demonstrates typical postoperative changes following division of the inferior mesenteric artery close to the aorta

The purpose of the supplementary arterial phase was to map the remaining mesenteric arterial anatomy in order to visualize whether the level of ligation of the IMA was located proximal or distal to the left colic artery. Intra-individual comparisons of the preoperative CT examinations were sometimes used to aid the evaluation. The level of ligation was determined as low if the left colic artery was preserved and high if it could not be identified (Figs. 1 and 2).

The CT-angiographies for all patients were evaluated independently by two radiologists: a senior consultant radiologist (FW) and a radiological trainee (PL). The imaging was assessed with regard to the ligation level of the IMA, without knowledge of the registered vascular ligation level in the SCRCR. Any disagreement between the radiologists was resolved by consensus. In addition, measurements were made of the length of the remaining vascular stump at the IMA origin at the postoperative imaging and of the length of the IMA trunk at the preoperative imaging; assessments were made using sagittal and coronal reconstructions and by means of maximal intensity projections. The IMA trunk was defined as the first part of the IMA, from the origin of the aorta to its first branch (most commonly the left colic artery). Finally, the distance from the origin of the IMA to the aortic bifurcation was evaluated on the axial reconstructions.

### Statistical analyses

With the radiological assessment as a reference to the registered ligation level in the SCRCR, we calculated sensitivity, specificity, and positive and negative predictive values as well as Cohen’s Kappa value with associated 95% confidence intervals (95% CI) [19, 20]. The Kappa value reflects the strength of the agreement. Kappa values < 0.6 are interpreted as poor to moderate agreement, 0.6–0.8 as good agreement, and ≥ 0.8 as almost perfect agreement. We also derived the Kappa value adjusted for prevalence bias [21], thus considering the effect of asymmetric distributions between ligation levels. In addition, we calculated the median and interquartile range (IQR) on the postoperative vascular stumps and the preoperative length of the IMA trunk, as well as the distance to the aortic bifurcation from the origin of the IMA. Secondary analyses were conducted, stratifying for center, minimally invasive surgery, and body mass index (BMI); the median was used to divide BMI into two similarly sized groups. Prior to study start, a power calculation was done: to achieve 90% sensitivity and specificity with a margin of error of ±10%, a sample size of 86 and 58 patients, respectively, was needed. All statistical analyses were performed using the statistical software STATA version 16.1 (StataCorp, Houston, TX, USA).

## Results

### Baseline characteristics

A convenience sample of 105 patients with operated rectal cancer was recruited into the study. From Umeå University and Örebro University hospitals, this constituted 72% and 21% of all operated patients during the study period, respectively. Eleven patients were excluded: the remaining IMA and branches were thrombosed in such a way that the radiological ligation level was not possible to determine (*n*=5), the CT-angiography was not performed at the same time as the postoperative follow-up control (*n*=4), severe renal impairment contraindicated a CT-angiography (*n*=1), and death before the planned CT-angiography (*n*=1). The final cohort constituted 94 patients, of whom 55 were men and 39 were women. The median age and BMI were 71 years and 25.8 kg/m^2^, respectively. The majority were operated with a minimally invasive approach, usually with an abdominoperineal excision. For clinicopathological details, see Table 1.

**Table 1:** Clinical data for all the included 94 patients operated for rectal cancer

Clinical variables	
	**Median (IQR)**
Age (years)	71 (65–75)
Body mass index (kg/m^2^)	25.8 (23.5–28.7)
	**Number (%)**
Sex	
Male	55 (58.5)
Female	39 (40.5)
Pathological tumor stage	
Complete response	8 (8.5)
I	25 (26.5)
II	20 (21.3)
III	36 (38.3)
IV	5 (5.3)
Surgical approach	
Open resection	38 (40.4)
Robot-assisted laparoscopy	53 (56.4)
Conventional laparoscopy	3 (3.2)
Operation type	
Anterior resection	33 (35.1)
Abdominoperineal excision	58 (61.7)
Hartmann’s procedure	3 (3.2)

*IQR* Interquartile range

### Registered ligation level of the IMA vs CT-angiography

The two radiologists were in initial agreement for 93 out of 94 cases; the single discrepant case was resolved by applying study criteria. The registered level of ligation of the IMA and the radiological assessment of the CT-angiographies were consistent in 77 of the 94 cases, demonstrating an 82% agreement. A total of 17 cases were non-matching: 8/29 radiological high arterial ligations were considered low by the operating surgeon, while 9/64 radiological low ligations levels were considered high by the surgeon (Table 2). In the main analysis, the unadjusted Cohen’s Kappa value was 0.58; with adjustment for prevalence bias, the Kappa value was 0.64. Percent agreement, sensitivity, specificity, and positive and negative predictive values, as well as Kappa values, are presented in Table 3.

**Table 2:** Registered ligation level of the inferior mesenteric artery and radiological assessment of ligation level on CT-angiography

		Radiological tie level		
		Low	High	Total
Registered ligation level	Low	56	9	64
	High	8	21	29
	Total	64	30	94

**Table 3:** Measures of agreement between registry and radiologist with 95% confidence intervals (CIs), using low ligation as reference

Measure	Point estimate	95% CI
Percent agreement	81.9%	74.0–89.8%
Sensitivity	86.2%	75.3–93.5%
Specificity	72.4%	52.8–87.3%
Positive predictive value	87.5%	50.6–85.3%
Negative predictive value	70.0%	76.8–94.4%
Cohen’s Kappa	0.58	0.40–0.76
Prevalence-adjusted Kappa	0.64	0.48–0.80

### Anatomy of the IMA

Preoperatively, the length of the IMA trunk was median 35 mm with a range of 16–80 mm (IQR 28–42 mm); the distance from the origin of the IMA to the bifurcation of the aorta was median 42 mm with a range of 2–88 mm (IQR 36–52 mm). Postoperatively, the median remaining IMA stump was 2 mm (IQR 0–6 mm) after high ligation and 44 mm (IQR 36–53 mm) after low ligation, respectively.

**Fig. 3:**
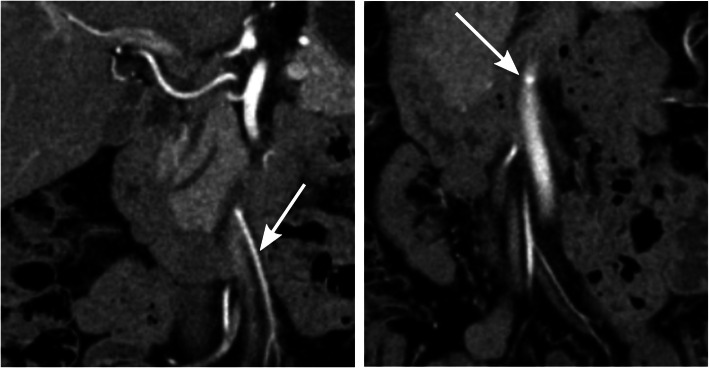
Example of where the ligation level was registered as low in the Swedish Colorectal Registry but where the radiological examination determined that the ligation level was high. In the preoperative image to the left, the inferior mesenteric artery (IMA) is indicated with an arrow. The postoperative image to the right demonstrates that all that remains of the IMA is a small bud-like contrast-filled bulge at the exit from the aorta

Typically, after low ligation, the IMA was moved laterally when compared to the preoperative examination, and the remaining left colic artery extended upward towards the remaining descending or sigmoid colon. After high ligation, there was usually a remaining small short stump of the IMA or a small bud-like bulge at the exit from the abdominal aorta (Fig. 3).

### Subgroup analyses

Secondary analyses were performed, stratifying for operating center (Umeå University Hospital or Örebro University Hospital), surgical approach (open or minimally invasive), and BMI (≤ 26 kg/m^2^ or > 26 kg/m^2^); for details, see Supplementary Tables 2 and 3. A discrepancy was demonstrated between the two study centers, whereas the measures of agreement were better at Örebro University Hospital. The agreement between the registered ligation levels and the radiological assessments was better for the high ligation group in laparotomy patients, and the reverse was found for those operated with laparoscopic surgery. Lower BMI was associated with a lesser agreement between surgeon and radiologist; further stratification by surgical approach and study center did not alter these results (data not shown).

## Discussion

This validation study indicates a discrepancy between the registered surgical ligation level of the IMA in the SCRCR and the assessed level of ligation on postoperative CT-angiography. The study also demonstrates that CT-angiography is a reliable validation tool, as the IMA and the left colic artery were clearly visualized and as it was possible to reliably assess the ligation level in the majority of cases. In this study, low ligations were more frequent than high ones, reflecting the operating traditions at the two centers. The agreement between the surgeons’ intraoperative and the radiological postoperative assessments was generally better in the low ligation group compared with the high ligation group. The Kappa value of 0.58 is difficult to assess, as this is affected by the fact that the distribution of high and low ligature is asymmetric [18, 19]. Taking this prevalence bias into consideration, the Kappa value becomes slightly better at 0.64, indicating a moderate to good agreement in the main analysis. These results correspond well to the sensitivity and specificity measures of 86% and 72%, respectively.

A potential limitation of this study is the possibility that the postoperative vascular topographic anatomy may change during the first postoperative year with vessel thrombosis and displacement, rendering the radiological assessment difficult. This should be of minor concern, as none of the analyzed patients with discordant ligation levels had totally thrombosed arteries, although five patients were excluded for that reason. A third limitation is that only two centers participated in the study, while the registration of the ligation level is nationwide, questioning the external validity of the current study; moreover, the inclusion rate was markedly different between these centers, possibly introducing selection bias. A fourth potential limitation stems from the radiological criteria applied, where a postoperative absence of the left colic artery denoted a low tie; other research [15] has indicated that some surgeons section both the superior rectal artery and the left colic, which could make the distinction between such a combination tie and a distal high ligation difficult. Finally, ascertainment bias might also be an issue, as it was possible to achieve only a convenience sample; nevertheless, it is difficult to fathom how such a bias could have a major impact on a study based on radiological assessments.

The major strength of the study is that it is prospective and based on two centers where surgeons independently registered the ligation levels of the IMA. Secondly, two radiologists assessed the imaging in an independent fashion, reaching an almost uniform interpretation on the radiological level of tie. Thirdly, the SCRCR has an excellent coverage, and none of the patients had to be excluded because the ligation level could not be found in the registry.

When comparing open surgical approach with minimally invasive surgery, the agreement between the registered ligation levels and the radiological assessments was better for the high ligation group in laparotomy patients. For those patients operated with laparoscopic surgery, the results were reversed, as the correlation in the low ligation group was better than in the high ligation group (Supplementary Tables 2 and 3). A general perception is that it is technically easier to perform a high ligation during laparoscopic rectal surgery; however, the results indicate that it is easier to at least estimate the level of ligation while performing open rectal surgery. Moreover, there was a discrepancy between the two study centers, where the measures of agreement were notably better at Örebro University Hospital, as compared to Umeå University Hospital. Barring error induced from the small sample size at the former center, this suggests that substantial variation might exist between centers considering the ligation level assessment (Supplementary Tables 2 and 3). Surprisingly, a lower BMI seemed to be associated with a lesser agreement between surgeon and radiologist; this was not driven by confounding from surgical approach or center and remains to be explained.

Previous studies of the surgically registered ligation level in the SCRCR have also shown uncertain reliability, although a comparison with CT-angiography has never been made before. Boström et al. compared operative notes with the registered ligation level in the SCRCR and found a good but not perfect match between them—85.5% agreement with Cohen’s Kappa 0.70—which is in line with our results. The authors argued that the demonstrated discrepancy was due to registration errors and difficulties of intraoperative assessment of the IMA anatomy [15]. The latter explanation is supported by Prevot et al. who investigated patients undergoing radical resection for sigmoid cancer [5], where a 41% agreement between surgeon’s assessment and findings on postoperative CT was demonstrated, without any influence by surgical approach, gender, or BMI. The surgeon underestimated the length of the remaining IMA in 70% of the cases, and thus perceived that the arterial ligation was conducted closer to the origin at surgery than was found on the postoperative CT. The above studies corroborate the findings of the current study that the vascular anatomy of the IMA is difficult to evaluate intraoperatively, perhaps in particular when performing minimally invasive rectal cancer surgery with high ligation. Moreover, a recent review and meta-analysis concluded that 1.2% of patients have an absent left colic artery, while also indicating that the left colic artery in about half of cases exhibit a fan-like spreading pattern along with the sigmoid arteries, making dissection in this area especially difficult [22]. Nevertheless, in the present study, there was no case of an absent left colic artery, judging from the preoperative imaging.

The literature for comparison of this study’s findings on the IMA anatomy is unfortunately scarce, which is partly explained by variable definitions of the IMA in the literature where it is found to be defined as either a) measured as the distance from the aortic origin to its first branch or b) measured as the distance from the aortic origin to its terminal branch [6]. Zhang et al. performed digital subtraction angiographies in 154 patients to explore the left-sided colorectal perfusion in non-operated patients and showed an average length of the IMA trunk (distance from aortic origin to the first branch) of 37 ± 15 mm [3]. Murono et al. also estimated the length of the IMA trunk in 471 non-operated patients using 3-dimensional CT-angiography, providing a median of 40 mm (range 10–82 mm). Both findings correspond well with the measurements of the IMA trunk and the IMA stump after low ligation, conducted in this study [16]. In addition, the preoperative distance from the origin of the IMA to the aortic bifurcation evaluated in the current study, median 42 mm, lies in the range of 30–60 mm as provided by earlier research [6], further validating our CT-angiography method.

This study shows that CT-angiography can be used to evaluate the reliability of the registered ligation level in the Swedish Colorectal Cancer Registry and that the correlation between the surgeons’ and the radiological assessments was moderate to good. However, the current study corroborates previous research that the intraoperative estimation of the ligation level of the IMA is difficult, suggesting also that the assessment might vary by surgical approach and center. To improve this intraoperative assessment, it is likely necessary to either refine the preoperative planning (e.g., using CT-angiography with reconstructed images) or routinely dissect the root of the IMA to completely visualize the arterial anatomy. For the moment, the provided validity estimates can be used to perform quantitative bias analysis in future registry-based studies, where misclassification of the ligation level might be of importance.

## Supplementary Information

**Additional file 1: Supplementary Figure 1**. The image used in the Swedish Colorectal Cancer Registry to indicate ligation levels. The inferior mesenteric artery (10), the left colic artery (11), and the superior rectal artery (12) are shown.

**Additional file 2.**

## Data Availability

The analyzed dataset used in the present study is available from the corresponding author on reasonable request.

## Abbreviations

CT: Computed tomography
IMA: Inferior mesenteric artery
SCRCR: Swedish Colorectal Cancer Registry
IQR: Interquartile range
BMI: Body mass index
