# Supplementary material for: Discrepancy between surgeon and radiological assessment of ligation level of the inferior mesenteric artery in patients operated for rectal cancer—impacting registry-based research and surgical practice
Source: World J Surg Oncol. 2021 Apr 13;19:115. doi: 10.1186/s12957-021-02222-5 (PMC8045315; doi:10.1186/s12957-021-02222-5)
Supplement: Supplementary file 2 — Additional file 2. [file 12957_2021_2222_MOESM2_ESM.docx]

## Supplementary Tables

**Supplementary Table 1.** Examination parameters and protocol for the computed tomography angiography of the mesenteric arteries.

| *Parameters* | GE LightSpeed VCT | Siemens definition AS | Siemens Definition Flash | GE RevCT |
| --- | --- | --- | --- | --- |
| Tube voltage (kV) | 100/ >90 kg 120 | Care kV^1^ | Care kV^1^ | 100/>90 kg 120 |
| Tube current min-max (mA) | 80–750/150–700 |  |  | 80–720 |
| Radiation dose automatic | Noise Index 38 | Quality ref. mAs 150 | Quality ref. mAs 210 | Noise Index 34 |
| Rotation time (s) | 0.5 | 0.5 | 0.5 | 0.5 |
| Pitch | 0.984:1 | 1.2 | 0.6 | 0.984:1 |
| Delay (s) | 8 | 5 | 8 | 8 |
| Bolus tracking | ROI renal level | ROI diaphragm level | ROI renal level | ROI renal level |
| Threshold value | 160 HU | 160 HU | 160 HU | 160 HU |
| Direction | craniocaudal | craniocaudal | craniocaudal | craniocaudal |
| Iterative reconstruction | ASIR 30% | SAFIRE 3 | SAFIRE 3 | ASIR-V 40% |
| Increment | 3/2.5 | 3/3 | 3/2.5 | 2.5/2 |
| Detector width (mm) | 38.4 | 38.4 | 38.4 | 40 |
| Reconstruction algorithm | Soft | I 41f | I 30f | Soft |
| Iodine contrast concentration | Omnipaque 350 mg/ml | Omnipaque 350 mg/ml | Iomerone 350 mg/ml | Omnipaque 350 mg/ml |
| Amount of iodine contrast | 0.4 g/kg | 0.35 g/kg | 0.4 g/kg | 0.4 g/kg |

^1^Care kV. Automatic adjustment of tube current depending on patient size and attenuation in the area of investigation (Siemens Healthineers, Erlangen, Germany). ASIR = adaptive statistical iterative reconstruction HU = Hounsfield unit. kV = kilovolt. mAs = milliampere second. ROI= region of interest. SAFIRE – sinogram affirmed iterative reconstruction. VCT – volume computed tomography Rev CT – revolution computed tomography.

**Supplementary Table 2.** Registered and radiological assessment of the ligation level of the inferior mesenteric artery, presented by subgroups.

| Subgroup |  |  | Radiological tie level | |
| --- | --- | --- | --- | --- |
|  |  |  | Low | High |
| Umeå University Hospital | *Registered ligation level* | Low | 34 | 9 |
|  |  | High | 7 | 19 |
|  |  |  |  |  |
| Örebro University Hospital | *Registered ligation level* | Low | 22 | 0 |
|  |  | High | 1 | 2 |
|  |  |  |  |  |
| Open surgical approach | *Registered ligation level* | Low | 21 | 2 |
|  |  | High | 4 | 11 |
|  |  |  |  |  |
| Minimally invasive surgical approach | *Registered ligation level* | Low | 35 | 7 |
|  |  | High | 4 | 10 |
|  |  |  |  |  |
| Body Mass Index ≤ 26 | *Registered ligation level* | Low | 27 | 7 |
|  |  | High | 7 | 8 |
|  |  |  |  |  |
| Body Mass Index > 26 | *Registered ligation level* | Low | 29 | 2 |
|  |  | High | 1 | 13 |
|  |  |  |  |  |

**Supplementary Table 3.** Measures of agreement between registry and radiologist with 95% confidence intervals (CIs), using low ligation as reference and presented by subgroups.

| Measure | Point estimate (95% CI) |
| --- | --- |
| Umeå University Hospital |  |
| Percent agreement | 76.8% (66.6% - 87.0%) |
| Sensitivity | 79.1% (64.0% - 90.0%) |
| Specificity | 73.1% (52.2% - 88.4%) |
| Positive predictive value | 82.9% (67.9% - 92.8%) |
| Negative predictive value | 67.9% (47.6% - 84.1%) |
| Cohen’s Kappa | 0.51 (0.30 – 0.73) |
| Prevalence-adjusted Kappa | 0.54 (0.33 – 0.74) |
| Örebro University Hospital |  |
| Percent agreement | 96.0% (87.7% - 100%) |
| Sensitivity | 100% (84.6% – 100%) |
| Specificity | 66.7% (9.43% - 99.2%) |
| Positive predictive value | 100% (15.8% - 100%) |
| Negative predictive value | 95.7% (78.1% - 99.9%) |
| Cohen’s Kappa | 0.78 (0.33 – 1.00) |
| Prevalence-adjusted Kappa | 0.92 (0.76 – 1.00) |
| Open surgical approach |  |
| Percent agreement | 84.2% (72.1% - 96.4%) |
| Sensitivity | 91.3% (72.0% - 98.9%) |
| Specificity | 73.3% (44.9% - 92.2%) |
| Positive predictive value | 84.6% (54.6% - 98.1%) |
| Negative predictive value | 84.0% (63.9% - 95.5%) |
| Cohen’s Kappa | 0.66 (0.40 – 0.92) |
| Prevalence-adjusted Kappa | 0.68 (0.44 – 0.93) |
| Minimally invasive surgical approach |  |
| Percent agreement | 80.4% (69.6% - 91.2%) |
| Sensitivity | 83.3% (68.6% - 93.0%) |
| Specificity | 71.4% (41.9% - 91.6%) |
| Positive predictive value | 58.8% (32.9% - 81.6%) |
| Negative predictive value | 89.7% (75.8 %- 97.1%) |
| Cohen’s Kappa | 0.51 (0.25 – 0.77) |
| Prevalence-adjusted Kappa | 0.61 (0.39 – 0.82) |
| Body Mass Index ≤ 26 |  |
| Percent agreement | 71.4% (58.3-84.5%) |
| Sensitivity | 79.4% (62.1%-91.3%) |
| Specificity | 53.3% (26.6%-78.7%) |
| Positive predictive value | 79.4% (62.1%-91.3%) |
| Negative predictive value | 53.3% (26.3%-78.7%) |
| Cohen’s Kappa | 0.33 (0.04-0.62) |
| Prevalence-adjusted Kappa | 0.43 (0.17-0.69) |
| Body Mass Index > 26 |  |
| Percent agreement | 93.3% (0.85%-100%) |
| Sensitivity | 93.5% (78.6%-99.2%) |
| Specificity | 92.9% (66.1%-99.8%) |
| Positive predictive value | 96.7% (82.8%-99.9%) |
| Negative predictive value | 86.7% (59.5%-98.3%) |
| Cohen’s Kappa | 0.85 (0.67-1.00) |
| Prevalence-adjusted Kappa | 0.87 (0.72-1.00) |
